# The impact of the Cyprus comprehensive smoking ban on air quality and economic business of hospitality venues

**DOI:** 10.1186/1471-2458-13-76

**Published:** 2013-01-27

**Authors:** Costas A Christophi, Martha Paisi, Despina Pampaka, Martha Kehagias, Constantine Vardavas, Gregory N Connolly

**Affiliations:** 1 Cyprus International Institute for Environmental and Public Health in association with Harvard School of Public Health, Cyprus University of Technology, 95 Eirinis Street, Rm 201, 3041, Limassol, CYPRUS; 2 Department of Environmental Health, Harvard School of Public Health, Boston, MA, USA; 3 Center for Global Tobacco Control, Department of Society, Human Development and Health, Harvard School of Public Health, Boston, MA, USA

**Keywords:** Environmental pollution, Tobacco smoke, Secondhand smoking, Smoking ban, Economic viability

## Abstract

**Background:**

Several countries, including Cyprus, have passed smoke-free legislations in recent years. The goal of this study was to assess the indoor levels of particulate matter in hospitality venues in Cyprus before and after the implementation of the law on 1/1/2010, evaluate the role of enforcement, and examine the legislation’s effect on revenue and employment.

**Methods:**

Several hospitality venues (n = 35) were sampled between April 2007 and January 2008, and 21 of those were re-sampled after the introduction of the smoking ban, between March and May 2010. Data on enforcement was provided by the Cyprus Police whereas data on revenue and employment within the hospitality industry of Cyprus were obtained from the Cyprus Statistical Service; comparisons were made between the corresponding figures before and after the implementation of the law.

**Results:**

The median level of PM_2.5_ associated with secondhand smoking was 161 μg/m^3^ pre-ban and dropped to 3 μg/m^3^ post-ban (98% decrease, p < 0.0001). Furthermore, in the year following the ban, the hotel turnover rate increased by 4.1% and the restaurant revenue by 6.4%; employment increased that same year by 7.2% and 1.0%, respectively.

**Conclusion:**

Smoke free legislations, when enforced, are highly effective in improving the air quality and reducing the levels of indoor PM_2.5_. Strict enforcement plays a key role in the successful implementation of smoking bans. Even in nations with high smoking prevalence comprehensive smoking laws can be effectively implemented and have no negative effect on accommodation, food, and beverage services.

## Background

Tobacco use is a major public health problem and the leading cause of preventable morbidity and mortality worldwide [1], while exposure to secondhand smoke (SHS) is a serious health hazard for non-smokers, especially children [2-4], SHS contains over 4,000 chemical compounds, including carcinogens, such as polycyclic aromatic hydrocarbons, aromatic amines, volatile- and tobacco-specific nitrosamines, as well as other toxic or irritating compounds, such as carbon monoxide, benzene, hydrogen cyanide, ammonia, and respirable particulate matter [5]. Over the past few years, an accumulating body of evidence has connected SHS with concerns about the health effects of indoor air-quality in public spaces, especially hospitality venues [6] and a number of studies have indicated that the introduction of smoking bans from all public spaces results in improved air-quality [7-9] and a significant drop in hospital admissions for myocardial infarction [10-17] and respiratory problems [18].

The initial success and prolonged maintenance of smoke free legislations differ significantly from country to country. For instance, after the implementation of a total smoking ban in Norway, a substantial reduction in airborne nicotine and total dust was observed in bars and restaurants (from 28.3 μg/m^3^ and 262 μg/m^3^, respectively, to 0.6 μg/m^3^ and 77 μg/m^3^) while the urinary cotinine levels also reduced in non-smokers (9.5 μg/m^3^ to 1.4 μg/m^3^) [8]. Similarly, in a study conducted in pubs before and two months after the implementation of Scottish legislation to prohibit smoking in substantially enclosed public places, indoor particle levels, as measured by PM_2.5_, reduced significantly from 246 μg/m^3^ to 20 μg/m^3^, an 86% reduction [19]. Secondhand smoke levels in Israeli pubs, bars and cafes also declined following the implementation of a non-comprehensive smoke free legislation (which extended existing restrictions on smoking in public places, including for the first time bars and cafes) and the noted reduction in PM_2.5_ reached 34% (from 245 μg/m^3^ to 161 μg/m^3^) [20]. The differences could be attributable to a number of factors, including the comprehensiveness of the law, where the responsibility for ensuring smoke free indoor public places was placed, the concurrent use population preparedness and signage [21], authority efficiency [22], social beliefs [23], tobacco industry interference [24], and the role of the non-smokers in actively demanding enforcement [25].

Besides its definite health related gains and the evidence from the majority of research that indicates that smoking bans have no dire economic effect on hospitality venues [26] arguments that smoking bans have detrimental effects on patronage and sales of the different establishments affecting both income and related employment are still expressed, mainly by owners of hospitality venues and other advocates for the rights of smokers, as noted in the literature [26,27]. In Cyprus, similar arguments, as in the rest of the world, are put forward (informal communication with the Health Committee of the Cyprus Parliament).

In Cyprus, cigarette smoking claims approximately 600 lives each year (out of an approximate total of 5000 deaths per year), while it has an annual direct economic burden exceeding 222 million Euros in healthcare expenditures and lost wages [28], not including the ramifications for passive smokers. (Though more recent data is not available, there is no evidence that these estimates have decreased.) Furthermore, Cyprus has one of the highest levels of cigarette consumption among EU member nations with prevalence rates being high among adults and youth alike, being 38.1% among adult males (daily smokers) and 35.7% among high-school boys (defined as having smoked on at least 1 of the last 30 days) and 10.5% among adult females (daily smokers) and 23.2% among high-school girls (defined as having smoked on at least 1 of the last 30 days) [29,30]. Unfortunately, smoking is still socially acceptable in Cyprus and several factors have been cited previously to be associated with the high prevalence of smoking among youth, including peers smoking, availability of pocket money, false consensus, and others [31].

Cyprus is a signatory of the Framework Convention on Tobacco Control (FCTC) and ratified it in 2003. One provision of the FCTC calls for restrictions on public smoking (Article 8, Section 2) [32]. So as to reduce smoking, protect non-smokers, and establish an environment that promotes health, Cyprus banned smoking in all enclosed public places and hospitality venues on January 1, 2010; more specifically, this was done under the new clauses added to the Protection of Health (Tobacco Control) Laws 2002–2009^a^ prohibiting smoking in all public places, including places of entertainment (restaurants, bars etc.), in all government buildings, public transport, and in private cars carrying any passenger under 16 years old. There is also a complete ban on advertising and promotion in mass media, such as television, cable television, radio, cinema, or other services of the information society, as defined in the Protection of Health (Tobacco Control) Laws 2002–2009 and the Cyprus Broadcasting Corporation Law of 1959–2010.

The present study assessed air quality, before and after the comprehensive law was passed, by measuring indoor levels of SHS within hospitality venues in Cyprus, examined the degree of compliance to the comprehensive smoking ban legislation and the role of enforcement authorities in this, and assessed the effect of the smoke free legislation on revenue and employment in the Cypriot hospitality industry, including restaurants, bars, nightclubs, and cafeterias.

## Methods

### Venues

A convenience sample of 35 venues was selected, with an effort made to include venues of different sizes and of different type, such as cafes, restaurants, hotels, bars, and nightclubs, selected from four different cities (Nicosia, Larnaca, Limassol, and Paphos) representing the larger 4 out of the 5 government controlled municipalities^b^; the distribution of selected venues by city is given in Table 1. The indoor air quality was measured between April 2007 and January 2008 and most venues were sampled during the late night hours, though there were some sampled in the early and late afternoons, at a time when, based on relevant experience of the researchers, these venues would be busy with patrons. Twenty-one of these venues (5 cafes, 5 restaurants, 6 restaurants/cafes, and 5 bars) were sampled again between March and May 2010, after the introduction of the smoking ban (due to budget constraints not all venues were re-sampled and no nightclubs were included). The study was exempt from any ethical review process as there were no human subjects recruited and no personal data were collected. Furthermore, no consent forms were obtained in order not to affect the behavior of owners and patrons.

**Table 1:** Venues sampled by city

**City**	**Pre-ban**	**Post-ban**
Nicosia	22	12
Larnaca	7	5
Paphos	4	4
Limassol	2	-
Total	35	21

### Air monitoring

To assess SHS and indoor air-quality, the levels of particulate matter with a mass median aerodynamic diameter less than 2.5 μm (PM_2.5_) attributable to SHS were measured. Particles of this size are released in large quantities from burning cigarettes and using standard methodology as previously described, can indicate exposure to SHS [5,7]. Briefly, a TSI SidePak AM510 Personal Aerosol Monitor (TSI, Inc., St. Paul, MN, USA) was used to measure the levels of PM_2.5_ in the selected locations. The device was set to record the level of PM_2.5_ every 10 seconds. In order to assess the fraction of PM_2.5_ that was attributable to indoor SHS, the levels of background air pollution (outdoor PM_2.5_) was subtracted from all findings, while a calibration factor of 0.32 and an air flow rate of 1.7 l/min were used. The protocol included spending at least 1 hour in the venue at a central location away from any other sources that could potentially affect the measurements (kitchen area, open windows, etc.) as well as spending at least 2 minutes outdoors before entering the venue and after exiting (to record background PM_2.5_ levels). Trained field workers patronized the venues in groups of two or more and air monitoring was conducted discretely to avoid disturbing occupants’ normal behavior.

### Enforcement information

The authority responsible for enforcing the smoke free legislation in Cyprus is the Police Department, which is part of the Ministry of Justice and Public Order. Police in Cyprus is authorized to issue €85 fines for those smoking in non-designated areas, while violators going to court could face up to a €2000 fine (this includes both the owner and the smoker). In addition, owners of establishments could face fines of up to €1000 if they fail to place highly visible no smoking signs where applicable.

### Revenue and employment in the hospitality industry

Information on turnover index (food and accommodation services) and employment within the hospitality industry in Cyprus were provided by the Cyprus Statistical Service. The hospitality industry economic activities are classified as accommodation services and food and beverages service activities. The major services in the category of food and beverages activities include restaurants and taverns, bars and pubs, nightclubs and discos, and cafeterias, which correspond to the main categories of venues where the PM_2.5_ measurements were conducted. We acquired data on the years prior to the introduction of the ban (2008 and 2009) as well as on the first year after the introduction of the ban, i.e. 2010, in order to make comparisons.

### Statistical analysis

All data were downloaded directly from the TSI SidePak AM510 Personal Aerosol Monitor using the corresponding software. Because of the skewed distribution of PM_2.5_ concentrations, median levels of PM_2.5_ before and after the smoking ban were calculated. The distribution of values of PM_2.5_ levels for the 21 venues that were measured both before and after the implementation of the law were compared using the Wilcoxon non-parametric test. All statistical tests reported are two-sided and a p-value < 0.05 is considered statistically significant. SAS 9.2 (SAS Institute, Cary, NC, USA) was used for the analyses.

## Results

### Indoor air quality

The median level of PM_2.5_ attributable to SHS among the 35 venues assessed before the comprehensive legislation was 184 μg/m^3^ (mean level of 295 μg/m^3^) in the venues that allowed smoking and 21 μg/m^3^ in the smoke-free venues. Levels of PM_2.5_ in the smoking venues ranged from 24 μg/m^3^ to 1413 μg/m^3^, while PM_2.5_ concentrations in the two non-smoking venues were zero and 42 μg/m^3^. (The latter value is not indicative of a typical smoke-free establishment since smoking was allowed in all surrounding spaces and the venue’s entrance was open to a lobby where smoking was allowed.)

**Figure 1:**
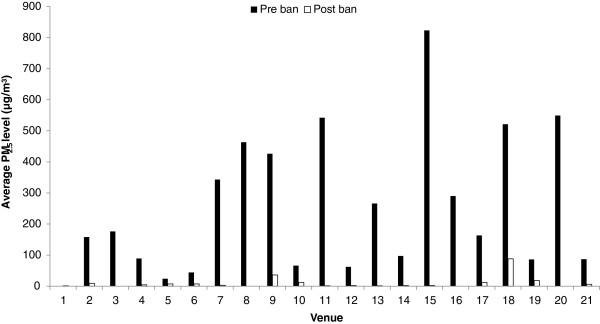
**PM**_**2.5**_**concentrations in individual venues.**

After the adoption of the smoke-free legislation in 2010, 21 venues were revisited; out of these, one used to be non-smoking prior to the legislation while the remaining ones were not. Figure 1 presents graphically the values of PM_2.5_ concentrations attributable to SHS measured before and after the implementation of the smoke-free legislation in these 21 venues; it is clear that post-ban there was a significant drop in PM_2.5_ levels in all venues in which smoking was previously allowed -- relatively higher values were observed in venue 18 were some patrons were seen smoking despite the law.

**Figure 2:**
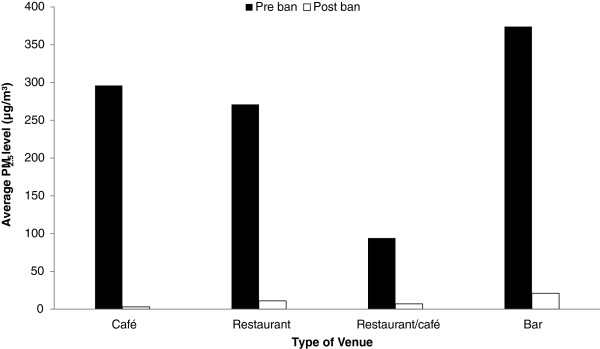
PM2.5 concentrations by type of venue.

The median level of PM_2.5_ attributable to SHS in these 21 venues was 161 μg/m^3^ (mean level of 251 μg/m^3^) pre-ban and dropped to 3 μg/m^3^ (mean level of 10 μg/m^3^) post-ban which corresponds to a 98% decrease (p < 0.0001). Results indicate that clubs and bars, on average, had the highest levels of PM_2.5_ before the implementation of the smoking ban; though no measurements were done in clubs post-ban, bars continued showing the highest levels. On average, the highest reduction in PM_2.5_ levels after the introduction of the ban was observed in cafés (99%), followed by restaurants (96%) and bars (94%), and then restaurants/cafes (93%) (Figure 2).

### Enforcement

During the first year since the introduction of the smoking ban (01/01/2010 to 31/12/2010) enforcement authorities carried out a total of 48900 inspections in all the government-controlled areas of Cyprus. As a result of these checks 6540 people were actually reported; 6449 on-the-spot fines were issued and 91 cases were sent to court. The highest absolute number of on-the-spot fines issued (n = 1799) and people reported (n = 1813) were in the category of pubs/bars, whereas traditional coffee shops had the highest number of cases that were sent to court (n = 26). The highest percentage of on-the-spot fines relative to the number of checks performed were observed in discos/clubs (39.4%), followed by other (34.0%), and betting shops (21.6%). From the total number of people reported (n = 6540), 2336 were owners/proprietors, 4175 were customers, and 29 were employees; the majority were Cypriots (n = 5515) while 1025 were foreign nationals; males were the majority (n = 5104) of offenders as opposed to women (n = 1436). The total figure includes 6 cases of minors (aged less than 18 years old).

### Economic revenue and employment before and after the comprehensive smoking ban

Based on data provided by the Cyprus Statistical Service [33], Table 2 presents the turnover index value for the accommodation and food and beverage activities, quarterly for 2008, 2009, and 2010, as well as the percentage change. The turnover indices of hotels and restaurants (food and beverage service activities) increased in 2010, i.e. during the first year of the smoking ban. The hotel turnover rate showed a downward trend from 2007 to 2009 but from 2009 to 2010 it increased by 4.1%. An even larger increase of 6.4% was observed in the restaurant industry. The turnover index of restaurants followed an increasing pattern in recent years with the highest values actually being reached in 2010.

**Table 2:** Turnover index before and after the implementation of the smoking ban in Cyprus in 2010

**Service**	**2008**	**2009**	**2010**	**Percentage Change 2010/2009**					**Percentage Change 2009/2008**				
	Jan- Dec	Jan- Dec	Jan- Dec	Jan- Mar	Apr- Jun	Jul- Sep	Oct- Dec	Jan- Dec	Jan- Mar	Apr- Jun	Jul- Sep	Oct- Dec	Jan- Dec
**Turnover index**													
Accommodation	116.3	100.5	104.6	+1.5	+0.4	+6.4	+6.1	+4.1	−19.4	−12.3	−13.9	−11.7	−13.6
Food and Beverage Activities	128.2	133.6	142.1	+3.0	+5.5	+7.6	+9.0	+6.4	8.8	6.0	1.3	1.6	4.2

Table 3 presents the number of people employed in the hospitality industry in Cyprus from January to December 2009 and the corresponding numbers for 2010 as obtained from the 2012 report on “Hotels and Restaurants Statistics” [34]. During 2010, the total number of employees in the hospitality industry increased by 3.7% compared to 2009. The accommodation sector experienced a rise of 7.2% and the food and beverages services an increase of 1.0%, indicating that there have been no net job losses in 2010 when the smoking ban was put in effect, as compared to 2009.

**Table 3:** Employment before and after the implementation of the smoking ban in Cyprus in 2010

**Service**	**2009**	**2010**	**Change (%)**
Accommodation	15517	16641	7.2
Food and Beverage	20571	20786	1.0
Total	36088	37427	3.7

## Discussion

Cyprus, with a high smoking prevalence rate of 38% among males 18 years and older and a tourism industry which includes visitors from both high and low smoking nations, presented a challenge in the implementation of the smoke-free law. However, overall, the results indicate that the implementation of the comprehensive smoking ban resulted in dramatic improvement on the indoor air-quality of hospitality venues. They also demonstrate that enforcement plays a key role in initiating and maintaining a smoke-free legislation. Supporting data also indicate that turnover indices for hospitality venues and employment in the hospitality industry do not suggest a negative economic impact after the implementation of the smoke-free legislation; on the contrary, they show an increase in the gross income of the hospitality industry. Though the reasons for this are not clear, it is suggesting that Cypriots may actually go out even more after the smoking ban was introduced, despite the signs of a world economic crisis at that point.

The significant changes in PM_2.5_ between pre- and post- ban measurements indicate dramatic improvement on indoor air-quality with PM_2.5_ levels attributable to SHS showing a 98% decline. This is in agreement with other studies that showed that enforcement of comprehensive smoking bans can have a dramatic effect on indoor air-quality, documenting drops in levels of PM_2.5_ ranging from 84-93% [7,19]. On average, levels of SHS were higher in bars than cafes or restaurants, which is in agreement with the Cyprus Police statements in regards to the on-spot-fines given, indicating that, within a year since the smoking ban came into effect, the majority of offenders (as absolute numbers of on-spot fines) were bar patrons, with 1799 people fined versus 422 in cafes and 220 in restaurants (out of a total number of 6540). This may in part be explained by the fact that in restaurants and cafes the presence of families and children are more common than bars.

The main opposition to smoke-free legislations comes from the tobacco companies, hospitality venue owners, and hospitality associations who argue that smoking bans have a deleterious economic effect on the hospitality industry and a dire effect on employment. Some anecdotal and newspaper reports, as well as some restaurant surveys, claim that restaurants suffer economic losses because of the implementation of the legislation, however, studies conducted by health advocates showed no adverse effect [26,35]. Scollo et al. [26] analyzed 97 studies that referred to economic effects of smoke-free legislations and i) reported that the studies with a negative effect had odds of using a subjective outcome 4.0 times the odds of those with no negative effects, with the vast majority (94%) of these sponsored by the tobacco industry; ii) stated that studies that did not conclude a negative impact were 20 times more likely to have been peer-reviewed; and iii) concluded that there is a strong association between tobacco industry support and studies that suggest negative economic impacts. In a different study, using data drawn from 30 communities of California and Colorado in the U.S.A., taxable restaurant sales and retail sales were compared between 15 communities that had adopted a smoke-free policy and 15 communities that had not adopted such policies and no negative economic impact was found [36]. Furthermore in Massachusetts, U.S.A., meal taxes and employment in food, drinking and accommodation services were used as economic indicators in assessing the state-wide anti-smoking law [37]; the study did not provide significant evidence that state-wide tobacco regulation had affected in any negative way the different economic indicators. Consistent with these findings is the report on “The Health and Economic Impact of New York’s Clean Air Act” where the sales tax receipts for bars, full service restaurants, and total retail establishments before and after the ban were examined and no difference was observed [38]. Similar studies were done in Canada [39], Norway [40], and others; findings from Canada showed no decrease in the sales of restaurants and bars, in New Zealand the hospitality sector experienced growth after the ban was introduced, while in Norway revenue was not found to change despite the low temperatures, especially during the winter months, which could act as an obstacle to outdoor smoking. Similarly, we observed no negative effects in the different indicators used to assess the effect of the smoking ban to the hospitality industry in Cyprus (employment and turnover index) despite the fact that in 2010 the world economy was affected by a crisis that had negative consequences on expenditures and tourism, an important driver of the economy in Cyprus.

Our study has the advantage that data were available from the same venues during both the pre- and post- the introduction of the smoking ban periods. This gave the opportunity to evaluate the direct impact of the smoking ban, eliminating the effects of any selection bias. Furthermore, we were able to use data directly provided by the Cyprus Police and the Cyprus Statistical Service in terms of both the enforcement of the law and the economic indices used, respectively. There are, however, some limitations in our study, such as the fact that a convenience sample was used; the pre- and post-measurements were done at different times (throughout the year for pre but only spring for post measurements); and there was no available data in terms of other important confounders. However, we do not believe that these factors would have an impact in our analyses, especially given the dramatic improvement observed.

The main factor that contributed to the successful implementation of the smoking ban during the first few months of 2010 seems to be the active enforcement by the police authorities. In general, active enforcement plays a key role in the implementation of such laws with the state making it clear to the public that authorities will enforce the specific law which is there to stay and it is not just ‘on paper’. However, sustaining compliance is a challenge that needs strong political will, active participation of enforcement authorities, and public embracing of the law.

## Conclusions

The present study underscores the importance of enforcing smoke-free laws and demonstrates that banning smoking completely in public places is highly effective in reducing the levels of indoor PM_2.5_ attributable to SHS. Moreover, the implementation of the smoke-free legislation in Cyprus was not found to affect either hospitality revenue or employment. This is a study that shows that nations with high smoking rates can effectively enforce clean indoor air laws and greatly improve air quality in public places, while at the same time not affecting economic business of the hospitality industry. It has yet to be determined, but in all likelihood the Cyprus law will help decrease smoking prevalence, similar to what has been observed in other nations such as Uruguay [41]. Based on the Cyprus experience, one of the countries in the European Union with high smoking rates, other nations with high or low smoking rates in Europe and elsewhere, can effectively pass and enforce clean indoor air laws.

## Endnotes

^a^ Περί Προστασίας της Υγείας (Έλεγχος του Καπνίσματος) Νόμοι 2002–2009 (Ε.Ε. Παρ.Ι(Ι), Αρ. 4214, 24/7/2009)

^b^ There are 6 municipalities in Cyprus, namely Nicosia, Limassol, Larnaca, Famagusta, Paphos, and Kyrenia but only 5 of them are either fully or partly under the control of the Government of Cyprus, with Kyrenia being fully occupied since 1974.

## Competing interests

The authors declare that they have no competing interests.

## Authors’ contributions

GNC, CV, MK and CAC conceived the study, participated in the design, and helped in the development of the methodology. CAC and DP performed the statistical analysis and CAC, MP, DP, and MK developed the first draft of the manuscript. All authors contributed towards the revision of the manuscript to its final version. All authors read and approved the final manuscript.

## Pre-publication history

The pre-publication history for this paper can be accessed here:

http://www.biomedcentral.com/1471-2458/13/76/prepub
